# Supplementary material for: Content-rich biological network constructed by mining PubMed abstracts
Source: BMC Bioinformatics. 2004 Oct 8;5:147. doi: 10.1186/1471-2105-5-147 (PMC528731; doi:10.1186/1471-2105-5-147)
Supplement: Additional File 2 — The original results of the above study (non-essential files are deleted to keep the file size under the limit set by BMC bioinformatics). [file 1471-2105-5-147-S2.bz2 › chilibotAdditionalFile2/dip30/374ID7680645E8297/html/FGF2_FGFR4.html]

 


 **FGF2** and **FGFR4** 
  
Found 52 abstracts in PubMed, retrieved 30.  
 

 What does Google say? 
 PDF only 
| .edu only 

---

**Interactive relationship** (e.g. stimulation, inhibition, etc)

**Neutral relationship**- **FGFR4**  transduction cascades were differently activated by FGF1 and  **FGF2** .  Ref: 11336793 Biochim Biophys Acta, 2001
- Cell free complexes of heparin and recombinant  **FGFR4**  bound FGF 1 and FGF 2  [ **FGF2** ]  equally.  Ref: 10336501 J Biol Chem, 1999
- ... FGF 2  [ **FGF2** ]  binds well to FGFR1, FGFR2, and  **FGFR4**  but interacts poorly with KGFR.  Ref: 10950949 J Biol Chem, 2000
- In contrast, FGF 2  [ **FGF2** ]  bound equally well to complexes of both FGFR1 and  **FGFR4**  formed with endothelial cell derived HSPG, but the endothelial HSPG was deficient for the binding of FGF 1 to both FGFR complexes.  Ref: 10336501 J Biol Chem, 1999
- Isolated heparan sulfate proteoglycan HSPG from liver cells in cell free complexes with  **FGFR4**  restored the specificity for FGF 1 and supported the binding of both FGF 1 and FGF 2  [ **FGF2** ]  when complexed with FGFR1.  Ref: 10336501 J Biol Chem, 1999
- This distribution pattern suggests that FGF 2  [ **FGF2** ]  in spermatogonia is involved in the autocrine and paracrine regulation of the proliferation and differentiation of spermatogonia and spermatocytes via the receptors FGFR 1, FGFR 3 and FGFR 4  [ **FGFR4** ] .  Ref: 9681690 Histochem Cell Biol, 1998
- The biological significance of VEGF, EGF, and bFGF  [ **FGF2** ]  is mediated by their receptors, which belong to the family of tyrosine kinase receptors Flt 1 VEGFR 1, KDR VEGFR 2, EGFR, FGFR 1, FGFR 2, FGFR 3, and FGFR 4  [ **FGFR4** ] .  Ref: 12500263 Microsc Res Tech, 2003

**Non-interactive relationship** (e.g. studied together, co-existance, homology, etc.)

- Furthermore, transcript levels of FGF receptor 1 FGFR1 and 4  **FGFR4**  isoforms, as well as of aFGF and bFGF  [ **FGF2** ]  were assessed by RT PCR. aFGF treated myotubes displayed increased expression of aFGF and bFGF  [ **FGF2** ] , suggesting a paracrine effect of exogenous aFGF.  Ref: 10631813 Differentiation, 1999
- Using L6E9 skeletal myoblasts, devoid of endogenous FGF receptors, which have been engineered to express either FGF receptor 1 FGFR1 or FGF receptor 4  **FGFR4** , we have demonstrated that both receptors, known to be expressed in skeletal muscle cell precursors, were able to mediate uPA induction by FGF 2  [ **FGF2** ] .  Ref: 9442043 J Biol Chem, 1998
- Immobilized  **FGFR4**  also bound FGF 8 besides FGF 1 and FGF 2  [ **FGF2** ] .  Ref: 10736564 Int J Biochem Cell Biol, 2000
- Early phosphorylation of ERK2 was blocked by the dominant negative form of growth factor bound protein 2 and Ras, for FGF1  **FGFR4**  and  **FGF2**   **FGFR4** .  Ref: 11336793 Biochim Biophys Acta, 2001
- Positive staining was also found for acidic and not basic fibroblast growth factor  [ **FGF2** ]  FGF and two of its high affinity receptors FGFR 1 and FGFR 4  [ **FGFR4** ] .  Ref: 9915531 Am Surg, 1999
- This study compares effects of chronic electrical stimulation on the expression levels of FGF 1, FGF 2  [ **FGF2** ]  and their receptors FGFRI,  **FGFR4**  in rat tibialis anterior TA muscle of hypothyroid rat, as well as in satellite cell cultures derived from normal rat TA and soleus SOL muscles.  Ref: 10653356 Differentiation, 1999
- The comparison between 5 d stimulated hypothyroid TA muscle and 5 d stimulated TA and SOL derived satellite cell cultures revealed differences in the expression of FGF 1 and FGF 2  [ **FGF2** ] , but similar expression levels of FGFR1 and  **FGFR4** .  Ref: 10653356 Differentiation, 1999
- The phosphatidylinositol 3 kinase inhibitors wortmannin and LY294002 only prevented the early ERK2 phosphorylation triggered by  **FGF2**   **FGFR4**  but not by FGF1  **FGFR4** .  Ref: 11336793 Biochim Biophys Acta, 2001
- The induction of uPA by FGF 2  [ **FGF2** ]  and serum in FGFR1 and in  **FGFR4**  expressing myoblasts required the mitogen activated protein kinase pathway, since treatment of cells with a specific inhibitor of the mitogen activated protein kinase extracellular signal regulated kinase 2 kinase, PD98059, blocked uPA promoter induction.  Ref: 9442043 J Biol Chem, 1998
- Furthermore, the FGFR 4  [ **FGFR4** ]  ligand, basic fibroblast growth factor  [ **FGF2** ]  bFGF  [ **FGF2** ] , enhanced erythroid cell proliferation induced by SCF or insulin, and thus modulated both erythroid proliferation and differentiation in vitro.  Ref: 10557077 Oncogene, 1999
- Stimulation of FGFR1 by FGF1 or  **FGF2**  and  **FGFR4**  by FGF1 induced a sustained phosphorylation of extracellular signal regulated protein kinase 2 and meiosis reinitiation.  Ref: 11336793 Biochim Biophys Acta, 2001
- In order to investigate which receptor s may mediate  **FGF2**  signaling and their role in regeneration, we have studied expression of the four fibroblast growth factor receptors, FGFR1, FGFR2, FGFR3 and  **FGFR4** , and of the spliced variants, sFGFR and KGFR, in the regenerating spinal cord of the adult urodele, Pleurodeles waltl, following tail amputation.  Ref: 12379240 Neuroscience, 2002
- In contrast,  **FGFR4**  stimulation by  **FGF2**  induced an early transient activation of ERK2 and no meiosis reinitiation.  Ref: 11336793 Biochim Biophys Acta, 2001
- However, in contrast to FGFR1, when recombinant  **FGFR4**  was expressed back in epithelial cells by transfection, it failed to bind FGF 2  [ **FGF2** ]  unless heparan sulfate was depressed by chlorate or heparinase treatment.  Ref: 10336501 J Biol Chem, 1999
- In order to investigate further the role of FGF signalling during mouse calvarial bone and suture development, we have performed detailed expression analysis of the splicing variants of Fgfr1 Fgfr3 and  **Fgfr4** , as well as their potential ligand  **Fgf2** .  Ref: 10751173 Development, 2000
